# Clinical diagnostic value of simultaneous amplification and testing for the diagnosis of sputum-scarce pulmonary tuberculosis

**DOI:** 10.1186/s12879-017-2647-7

**Published:** 2017-08-05

**Authors:** Liping Yan, Qing Zhang, Heping Xiao

**Affiliations:** Department of Tuberculosis, Shanghai Pulmonary Hospital, Tongji University School of Medicine, 507 Zhengmin Road, Shanghai, 200433 People’s Republic of China

**Keywords:** SAT-TB assay, Pulmonary tuberculosis, Culture test, Smear test, BALF

## Abstract

**Background:**

Since 20% of pulmonary tuberculosis (PTB) patients are asymptomatic, the early detection of PTB is a challenge particularly in sputum-scarce patients and diagnostic accuracy based solely on clinical characteristics and chest X-ray/CT scans are not always satisfactory. The AmpSure simultaneous amplification and testing method for the detection of *Mycobacterium tuberculosis* (SAT-TB assay) is an alternative approach to diagnose PTB. In the present study, we analyzed the usefulness of the SAT-TB assay for PTB diagnosis in sputum-scarce patients.

**Methods:**

A total of 840 patients were prospectively enrolled for PTB diagnosis with bronchial alveolar lavage fluid (BALF) used as the samples for the SAT-TB assay. Of these, 536 had a definite diagnosis of PTB confirmed by positive microbiology culture, or clinical diagnosis of active PTB following anti-TB treatment with a favorable response.

**Results:**

The SAT-TB assay showed a 76.44% agreement with the culture test. The sensitivity and specificity of the SAT-TB assay were 50.75% and 94.73%, respectively. The sensitivity of SAT-TB was significantly higher than that of BALF cultures (21.64%) (X^2^ = 49.1503; *P* < 0.001) and smears (4.48%) (X^2^ = 175.2315; *P* < 0.001). The specificity of SAT-TB was slightly lower than that of BALF cultures (98.25%) (X^2^ = 2.0727; *P* = 0.150) and smears (98.25%) (X^2^ = 2.0727; *P* = 0.150). The accuracy rates were 63.87% for SAT-TB, 44.50% for BALF cultures and 29.84% for BALF smears.

**Conclusion:**

The high accuracy of the SAT-TB assay indicated that active PTB is present and anti-TB treatment is strongly recommended regardless of smear and culture test results for sputum scarce active PTB suspected patients when BALF SAT-TB is positive.

## Background

Tuberculosis (TB) now ranks alongside HIV as a leading cause of death. In 2015, 1.4 million people died from TB and 10.4 million people were estimated to have fallen ill with TB worldwide. However, only 3.0 million were bacteriologically confirmed [1] and the prevalence of asymptomatic PTB patients has been reported to be as high as 49% in China [2].

Bacteriological culture is considered the reference gold standard for detecting TB, but suffers from the disadvantages that results take weeks to obtain and testing requires a well-equipped laboratory and highly trained staff [3]. Smear microscopy remains the most widely used tool for TB diagnosis in low- and middle-income countries. However, its sensitivity is limited to more than 10,000 organisms/mL of sputum [4] and the test cannot distinguish between *Mycobacterium tuberculosis* complex (MTBC) and non-*tuberculos mycobacteria* (NTM).

A growing number of rapid and more sensitive tests for TB based on molecular methods have become available to complement existing conventional tests [5–7]. Simultaneous amplification and testing methods for detection of MTBC (SAT-TB assay) is a novel molecular technique intended for point-of-care testing for TB. It combines the technologies of nucleic acid isolation, real-time fluorescence simultaneous isothermal RNA amplification and testing with fluorescence-labeled hybridization probes. It is fast, simple and has good reproducibility [8]. Previous studies of the SAT-TB assay that focused on its accuracy with sputum samples demonstrated that its overall sensitivity for the diagnosis of PTB was 67.7% (range 39.2% to 93%) [9, 10].

Early detection of PTB is a challenge in sputum-scarce patients [11]. The diagnosis of TB is usually dependent on scoring systems based on clinical characteristics and chest X-ray/CT scans. Several previous studies evaluated the diagnostic accuracy, but found that it was not satisfactory [12–16]. The poor specificity of the clinical characteristics and X-ray/CT screening may result in people without TB being given treatment for TB as well as under-diagnosis of true TB cases. This often leads to irreversible lung damage in infected individuals and contributes in part to the risk of transmission of the disease.

Fiberoptic bronchoscopy (FOB) is an alternative option to provide respiratory specimens for diagnosis, particularly from sites which are suspected by radiological findings to be involved in PTB after sputum expectoration has continually failed because of lacking sputum [17–19].

We therefore designed the current prospective, controlled study using BALF specimens from suspected active PTB patients to evaluate the clinical diagnostic performance of SAT-TB assays in sputum-scarce PTB individuals after we have tried sputum induction in patients not able to expectorate a sputum in the resource-limited high-burden settings of China.

## Methods

### Patients

The Ethics Committee of the Shanghai Pulmonary Hospital approved this prospective study and written informed consent was obtained from each participant before enrollment.

All adult patients admitted to Shanghai Pulmonary Hospital were screened between April 2014 and January 2015. Inclusion criteria were: suspected active PTB; age ≥ 17 years; no previous history of anti-TB treatment; inability to provide sputum for examinations; negative HIV status. Exclusion criteria were: inability to tolerate bronchofibroscopy; no finalized diagnosis after examination and treatment (obscure diagnosis). When FOB was performed, BALF specimens from the affected lung were acquired for acid-fast bacilli (AFB) smear, mycobacterial culture and SAT-TB assays.

A standard questionnaire was completed by each patient before enrollment, including basic demographic data, history of TB contacts, previous TB, current TB symptoms, anti-TB treatments, as well as underlying diseases and concurrent therapies.

All patients to be evaluated for active TB were tested using the SAT-TB assay, AFB smear test, as well as culture tests at enrollment, in addition to physical, pathological and radiographic examinations.

Active PTB was confirmed when radiographic or chest computed tomography (CT) manifestations were in accordance with the PTB pattern and accompanied by one of the following criteria at enrollment or later during the study period: bacteriological diagnosis based on positive MTBC in cultures and pathological diagnosis of PTB based on analyses of AFB in resected lung tissues.

In patients who did not meet one of the above two criteria, clinical diagnosis was further confirmed by meeting all of the following categories: signs or symptoms of PTB and typical manifestation of TB on chest CT scans; received anti-TB medication for 2 months with a favorable response, based on improved signs or symptoms and chest CT results. For these cases, chest CT scans were reviewed every 2 months until the patients had been treated for 6 months.

At the end of the study, each patient was classified into one of three predefined clinical categories namely active PTB, non-TB pulmonary disease or without a final diagnosis (with obscure diagnosis or the patients lost to follow-up) by clinical experts who were blinded to the results of the SAT-TB assays.

### Fiberoptic bronchoscopy

Bronchoscopy procedures were performed according to our institute’s infection regulation and manufacturer’s instruction guidelines (BF-1 T260, Olympus, Tokyo, Japan). Inspectors wore N95 masks, goggles and gowns during the procedure. The bronchoscopy room was equipped with negative pressure isolation and an air disinfection system.

### Microbiological identification

#### Acid-fast bacilli smears and culture assays

BALF specimens were routinely tested by AFB smears and cultures with Lowenstein–Jensen medium, as well as with the Bactec MGIT 960 System (Becton Dickinson Diagnostic Systems, Sparks, MD) by following the standard procedure of the manufacturer [20]. Each specimen of approximately 5 ml was decontaminated using the N-acetyl-L-cysteine (NALC)–NaOH method [21]. The final concentration of NaOH is 4%. The samples exposed to NaOH for 15–20 min. The processed sediment was washed once using a sterile 0.9% NaCl solution and resuspended in 1.5 ml sterile 0.9% NaCl solution; three separate 500-μl aliquots were prepared in 1.5-ml tubes for the SAT-TB and Bactec MGIT 960 culture tests. The detection threshold of a positive smear was smear positive grade 1. All tests were performed at the tuberculosis reference laboratory in Shanghai Pulmonary Hospital, and quality controls were routinely performed. *M. tuberculosis* H37Rv (ATCC 27294) and 20 reference strains were gifts of the National Tuberculosis Reference Laboratory (Beijing, China).

#### Simultaneous amplification and testing method for detection of *Mycobacterium tuberculosis* (MTBC)

To process the clinical samples for the SAT-TB assay, the aliquoted processed sediments were centrifuged, the supernatants were discarded, and 50 μl TB dilution solution (10 mM sodium citrate, pH 8.0) was added to each tube and vortexed. Each sample was sonicated for 15 min at room temperature in a water bath sonicator (Shanghai Sheng-Yan Ultrasound Machines Co. Ltd., Shanghai, China) at 300 W and then centrifuged, and the supernatant was used in the SAT-TB assay. The live M. tuberculosis attenuated strain H37Ra (ATCC 25177) was used as a positive control, and the negative control was double-distilled water. For the assay, 2 μl of processed supernatant and 30 μl of a reaction solution containing 40mMTris-HCl, pH 8.1, 8mMMgCl2, 25mMNaCl, 2 mM spermidine hydrochloride, 5 mM dithiothreitol, 80 μg/ml bovine serum albumin, 200 μM dATP, 200 μM dTTP, 200 μM dGTP, 200 μM dCTP, 1 mM ATP, 1 mM GTP, 1 mM CTP, 1 mM UTP, and 0.5 mM (each) primers and probe were prepared in a 200-_l PCR tube. The mixture was preincubated at 60 °C for 10 min, followed by 42 °C for 5 min, and then a 10-μl aliquot containing 2000 units M-MLV reverse transcriptase and 2000 units T7 RNA polymerase (RD Bioscience, Inc., San Diego, CA) was added and gently mixed. The reaction mix was then placed immediately into a model 7500 real-time PCR system (Applied Biosystems Inc., Foster City, CA). RNA isothermal amplification was conducted at 42 °C for 1 min, with a total of 40 cycles, and FAM fluorescence data were collected after each cycle of amplification. Specimens with cycle thresholds (CT) of≦40 were classified as TB positive. The above processes were performed in a biological safety cabinet (class II B2) in a biosafety level PII laboratory. RNase-free tubes and tips were required for the SAT-TB assay.

### Statistical analysis

The data were entered into a computer and analyzed using SPSS for Windows (Version 18.0:, SPSS Inc., Chicago). Sensitivity, specificity, positive and negative predictive values, as well as accuracy rates of the SAT-TB assay, BALF smear and cultures were calculated. The categorical variables were analyzed using Fisher exact or Pearson X2 tests where appropriate and 2-tailed tests were used. The concordance of agreement between SAT-TB assays and smear and culture data was assessed using Cohen’s kappa test (k > 0.75, excellent agreement; 0.4 < k < 0.75, moderate agreement; and k < 0.4, poor agreement). *P* values <0.05 were considered to be statistically significant. Receiver operating characteristic (ROC) curve analysis was performed to determine the diagnostic power of SAT-TB assays.

## Results

### Study patients and results of diagnostic methods

A total of 840 hospitalized sputum-scarce patients with suspected active PTB were prospectively enrolled into the study. Forty patients were excluded because of obscure diagnosis and 36 patients were excluded because of loss to follow up. Finally, the data from 764 patients were analyzed. According to comprehensive evaluations and follow-ups, 536 patients (70.16%) were diagnosed with PTB and 228 patients (29.84%) with non-TB pulmonary disease (68 were confirmed by microbiology and 160 subjects by clinical diagnosis). Among the 536 active PTB patients, 116 (21.64%) were confirmed by microbiology and 420 subjects (78.36%) by clinical diagnosis (Fig. 1).

**Fig. 1:**
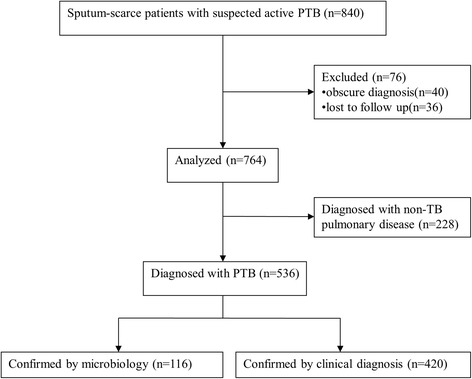
Flow chart of the study

### Detection of PTB by smear and culture tests, SAT-TB assays or by clinical examination

Among the 120 PTB patients who had positive BALF cultures, 108 also had positive SAT-TB results (108/120; 90.00%). In the 20 subjects whose BALF smears and cultures were both positive, the SAT-TB positivity rate was 100% (20/20). Notably, SAT-TB detected 164 PTB cases (39.05%), which were negative for BALF culture tests (Table 1).

**Table 1:** Overall results of different diagnostic tuberculosis evaluations

Characteristic	PTB (*n* = 536)	Non-TB disease (*n* = 228)
Male sex, No. (%)	372 (69.40)	156 (68.42)
Mean age (years/range)	41 (19–64)	42 (20–64)
SAT-TB results	272	12
PTB diagnosis sources		
Microbiology	116	68
Positive SAT-TB	108	0
Positive culture	116	0
Positive smear	20	0
Clinical diagnosis	420	160
Positive SAT-TB	164	12
Positive culture	0	4
Positive smear	4	4

### Performance of three microbiological assays

The sensitivity of BALF SAT-TB (272/536, 50.75%) for the diagnosis of sputum-scarce PTB was significantly higher than that of BALF cultures (116/536, 21.64%) (*P* < 0.001) and smears (24/536, 4.48%) (*P* < 0.001). There was a significant difference between BALF cultures and smears (*P* < 0.001). The specificity of SAT-TB (216/228, 94.73%) for the diagnosis of PTB was slightly lower than that of BALF cultures (224/228, 98.25%) (*P* = 0.150) and smears (224/228, 98.25%) (*P* = 0.150). For the 116 BALF culture positive PTB patients, the sensitivity of the SAT-TB assay was 93.10% and was significantly higher than that of BALF smears (17.24%, *P* < 0.001). Of the 420 BALF culture negative PTB patients, the sensitivity of the SAT-TB assay was 39.05%, which was significantly higher than that of BALF smears (0.95%, *P* < 0.001) (Table 2).

**Table 2:** Comparison of SAT-TB, BALF smear and culture results

	Sensitivity	Specificity	PPV	NPV	Accuracy Rates
SAT-TB	50.75%	94.73%	95.77%	45.00%	63.87%
BALF culture	21.64%	98.25%	96.67%	34.78%	44.50%
BALF smear	4.48%	98.25%	85.71%	30.43%	29.84%

*BALF* Bronchial Alveolar Lavage Fluid, *PPV* Positive Predictive Values, *NPV* Negative Predictive Values

### Consistency between SAT-TB assays and bacterial cultures for the detection of mycobacterium tuberculosis complex

The agreement between the tests was measured by Cohen’s kappa (κ) statistics. The SAT-TB assay and BALF cultures showed a moderate concordant result (κ = 0.413, *P* < 0.001), indicating a suboptimal agreement between the two tests.

### Establishment of receiver operating characteristic (ROC) curve

The area under ROC curve (AUC) of BALF smears and cultures and SAT-TB assays was 0.51 (95% CI 0.47–0.56), 0.60 (95% CI 0.56–0.64) and 0.73 (95% CI 0.69–0.76). SAT-TB assays had significant value on diagnosis of TB (*P* = 0.001) (Fig. 2).

**Fig. 1:**
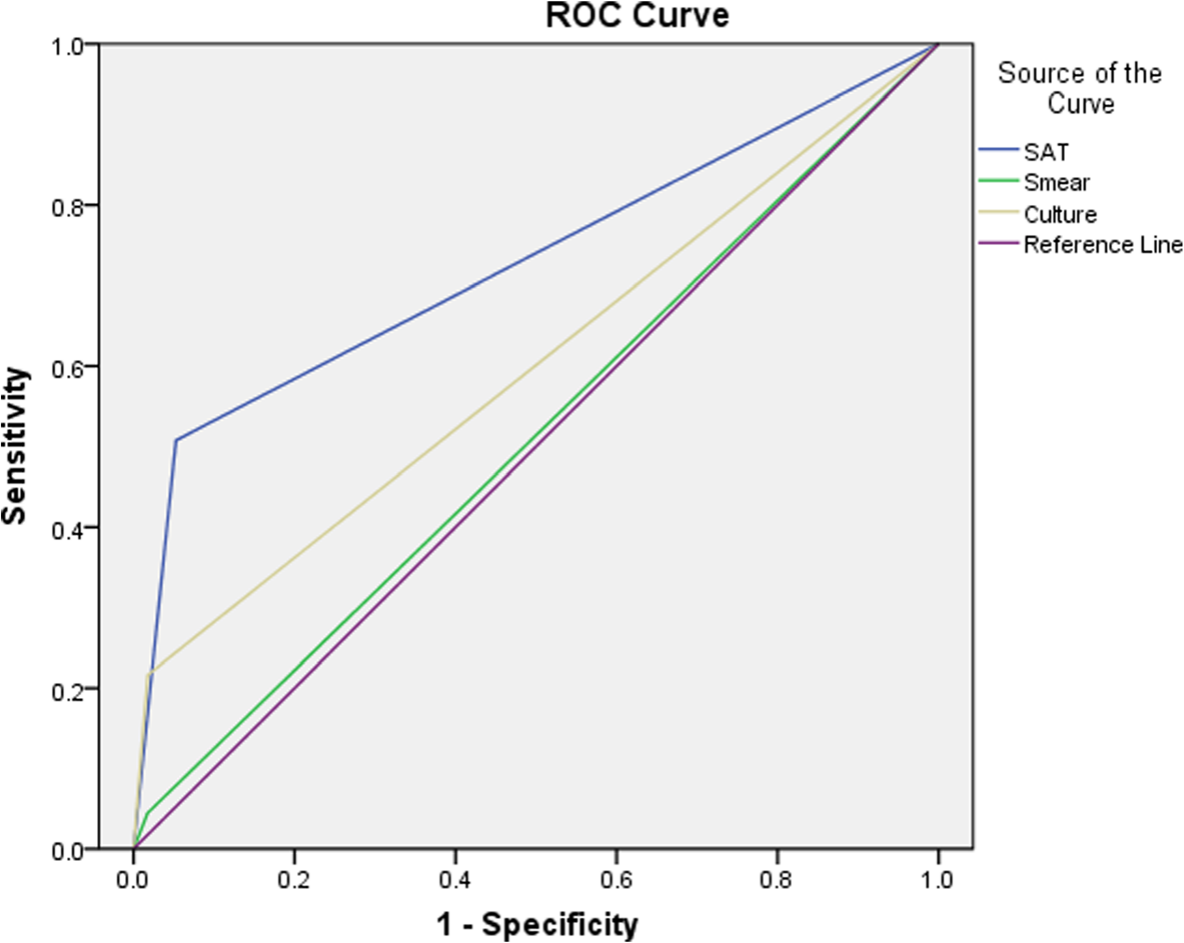
ROC Curve

## Discussion

Mycobacterium culture is the gold standard for confirming TB but can take up to 8 weeks to obtain the results, rendering this diagnostic method inconvenient in routine clinical practice. Despite the fact that it is invasive, FOB is considered useful for the diagnosis of PTB when sputum samples cannot be obtained. In our prospective study, we evaluated the clinical value of BALF SAT-TB assays for the diagnosis of active PTB in sputum-scarce PTB suspects in TB endemic settings.

Technological advances in nucleic acid amplification have led to breakthroughs in the early detection of PTB. The high cost of the GeneXpert (Cepheid, Sunnyvale, CA) assay and the requirement of expensive specialized equipment for the MTD test (Gen-Probe; San Diego, CA, USA) prevent these assays from being widely adopted in resource-limited settings [22, 23]. In China, the cost for a SAT-TB assay is $30 and under medical reimbursement coverage, whereas the cost of a GeneXpert is $100 and not covered by public medical insurance. The SAT-TB assay can be performed on real-time PCR instruments, which found in most clinical laboratories. Results from BALF with the SAT-TB assay are usually received within 120 min. SAT-TB assay can be performed 7 days a week.

The sensitivity of the SAT-TB test in our study was 50.75% vs 21.64% for culture detection, which was lower than the 90% reported by Fan et al. (2014) in whose report the SAT-TB sensitivity was found to be only slightly different from culture tests [10]. It is worth mentioning that we choose the sputum scare patients, not all TB patients and SAT-TB detected 164 PTB cases (39.05%), which were negative for BALF culture tests in this study.

The proposed reasons for sensitivity restrictions are unequal distribution in the test suspension and the presence of inhibitors of enzymatic amplification [23, 24]. In addition, our research differs from previous work in terms of its design. We enrolled sputum-scarce patients with suspected active PTB into the study, but the lesions in these patients are generally mild and peripherally localized. Therefore, the SAT-TB sensitivity limitation in our study might also be due to low numbers of Mycobacteria because of incomplete access to the infected areas.

The current findings suggest that the SAT-TB assay, which showed a 0.413 agreement with culture methods, is more accurate for detecting MTBC in BALF than smear and culture tests. As the SAT-TB assay results had a high PPV (95.77%) and can be accomplished within 120 min, when a BALF sample is positive regardless of the results of smear, it is recommended that anti-TB treatment be started in clinically suspected PTB without waiting for culture results (6–8 weeks). If a BALF sample is SAT-TB assay negative and smear positive, NTM infections could be a possible result of the diagnosis and an NTM related test is recommended. Two patients with NTM lung disease were identified as positive by sputum smears but were negative according to the SAT-TB assay results in the present study.

## Conclusion

In summary, we found that the SAT-TB assay of BALF specimens could increase the TB diagnostic yield significantly and is more useful than a BALF smear test. It is an accurate diagnostic method for PTB, especially in developing countries because of its low cost and rapidity. In contrast, its low negative predictive value limits its usefulness to exclude the diagnosis of PTB and therefore SAT-TB-negative specimens still require conventional culture tests and specification. In addition, false-positive and false-negative results are unavoidable with the current technology. Further multicenter studies and large cohort studies are needed to confirm unequivocally the findings of the present study.

## Abbreviations

BALF: bronchial alveolar lavage fluid
CT: computed tomography
FOB: Fiberoptic bronchoscopy
MTB: Mycobacterium tuberculosis
NTM: non-tuberculos mycobacteria
PTB: pulmonary tuberculosis
SAT-TB assay: The AmpSure simultaneous amplification and testing method for the detection of Mycobacterium tuberculosis
SD: standard deviation
TB: Tuberculosis
